# AGE-FRIENDLY RESEARCH: EDUCATION AND TOOLS TO ENHANCE INCLUSION OF OLDER ADULTS IN RESEARCH

**DOI:** 10.1093/geroni/igae098.1170

**Published:** 2024-12-31

**Authors:** Allison Lindauer, Bryanna De Lima, Elizabeth Eckstrom

**Affiliations:** Oregon Alzheimer’s Disease Research Center, Oregon Health & Science University, Portland, Oregon, United States; Oregon Health & Science University, Portland, Oregon, United States; Oregon Health & Science University, Portland, Oregon, United States

## Abstract

Many common diseases disproportionately affect older adults but research studies under-represent older adults due to recruitment and retention barriers. Geriatric research specialists have designed frameworks and tools to address these challenges but these are not widely disseminated. Based on these frameworks and tools, our team designed an Age Friendly Research synchronous, interactive 6-session webinar series and seven Age-Friendly research tools for research team members at our institution. We recruited 40 research-level employees to participate in the webinar series from October to November 2022 and 21 to pilot test the tools. Webinar participants completed pre- and post-program surveys and post-webinar session surveys in REDCap. Participants who pilot tested the tools completed up to 4 monthly REDCap surveys. Surveys included quantitative (e.g., Likert scale, dichotomous, and 10-point ratings) and open-ended responses related to strengths, limitations, areas for improvement, and intention to implement newly learned information. Webinar participants expressed positive feedback stating they would likely use what they learned and recommend to others. Strengths of the webinar were practical tips, useful tools, and real-world examples. However, more information on industry-sponsored trials was requested. The most utilized tools were the communication guide and Age-Friendly research checklist. Participants appreciated the ease of use, webinar training, and supportive teams for tool implementation but identified lack of time and age-appropriate study populations as key barriers. Overall, the webinar and tools were well-received by participants. Sharing the webinar and tools with others could lead to more Age-Friendly research and improve the experience for researchers and older adults alike.

